# Methylation of histone H3 at lysine 23 in meiotic heterochromatin

**DOI:** 10.1186/1756-8935-6-S1-O13

**Published:** 2013-03-18

**Authors:** Romeo Papazvan, Ekaterina Voronina, Jessica R Chapman, Tonya M Gilbert, Elizabeth Meier, Jeffrey Shabanowitz, Donald F Hunt, Yifan Liu, Sean D Taverna

**Affiliations:** 1 Department of Pharmacology and Molecular Sciences, and the Center for Epigenetics, The Johns Hopkins University School of Medicine, USA; 2 Department of Molecular Biology and Genetics and Howard Hughes Medical Institute, Center for Cell Dynamics, The Johns Hopkins University School of Medicine, USA; 3 Department of Chemistry, University of Virginia, USA; 4 Department of Pathology, University of Virginia, USA; 5 Department of Pathology, University of Michigan Medical School, USA

## 

Heterochromatin and its associated histone modifications are important for repressing transcription and maintaining chromosomal integrity during meiosis and mitosis. The complex repertoire of histone modifications that decorate heterochromatin has yet to be fully characterized, in part because most eukaryotic cells have a single nucleus where distinct chromatin types are intermingled on contiguous stretches of chromosomes. To obtain highly-purified heterochromatin, we turned to the model organism *Tetrahymena thermophila*, which has a biochemically separable heterochromatic micronucleus. We characterized combinatorial histone modifications on heterochromatic H3 from *Tetrahymena* and identified species of H3 dually-modified by both H3K23me3 and H3K27me3 as a previously unreported binary ‘mark’ specific for heterochromatin. Furthermore, H3K23me3 levels dramatically increased during meiosis in *Tetrahymena* micronuclei, *C. elegans*, and mice, suggesting this histone ‘mark’ plays a conserved role in germline development. Lastly, disrupting the H3K23 methyltransferase in *Tetrahymena* caused a lag in meiotic progression. Together, our data suggests H3K23me3 is a conserved heterochromatic histone PTM strongly associated with meiosis, and misregulation of this modification may be linked to problems with reproductive fitness and development.

